# GaN-based mini-LED matrix applied to multi-functional forward lighting

**DOI:** 10.1038/s41598-022-10392-9

**Published:** 2022-04-19

**Authors:** Quang-Khoi Nguyen, Yi-Jou Lin, Ching Sun, Xuan-Hao Lee, Shih-Kang Lin, Chi-Shou Wu, Tsung-Hsun Yang, Tian-Li Wu, Tsung-Xian Lee, Chao-Hsin Chien, Yeh-Wei Yu, Ching-Cherng Sun

**Affiliations:** 1 Department of Optics and Photonics, National Central University, ChungLi, 32001 Taiwan; 2 International College of Semiconductor Technology, National Yang Ming Chiao Tung University, HsinChu, 30010 Taiwan; 3 Graduate Institute of Color & Illumination Technology, National Taiwan University of Science and Technology, Taipei, 10607 Taiwan; 4 Department of Electrophysics, National Yang Ming Chiao Tung University, HsinChu, 30010 Taiwan

**Keywords:** Engineering, Optics and photonics

## Abstract

In this paper, we propose and demonstrate to use of a single reflector with 68 segments to project vehicle low beam and high beam with the use of a GaN-based mini-LED matrix, which is a 5 × 6 LED die array. The design of the reflector is based on light field technology in considering etendue from the light source across the segments. The group of the segments with smaller etendue from the LED dies in the bottom 2 rows are used to project low beams. When the other LED dies are turned on, the reflector will project light upward and form the high beam. The selection of the turn-on LED dies in the mini-LED matrix can adjust the width of the illumination pattern so that an adaptive low/high beam can be performed. Besides, to extend the functionality of the headlamp module, we propose to dispense IR phosphor on LED dies in the high-beam zone of the GaN-based mini-LED matrix. Thus the vehicle can emit IR high beam, which can be imaged through a camera and can be incorporated with machine vision for an autonomous vehicle without using a complicated adaptive headlight to avoid glare. The proposed multi-function in spatial and spectral domains will be helpful to various applications with use of a mini-LED matrix.

## Introduction

Vehicle forward lighting, one of safety issues, provides clear vision to a driver, free of glare to people on the roadway, and avoids light pollution no matter what the street light is^1–12^. The forward lighting includes low beam, high beam, and fog light. The low beam is to support the least illumination for the driven vehicle, and a clear or high-contrast cutoff line is necessary to reduce glare. High beam is long-range illumination to a driver, but it can be used only when there is no people ahead of the driven vehicle. The fog light is used only in the foggy condition, and the illumination is for the close area^13–16^. For a driver, high beam is the best solution to provide clear vision, but it can be used in a very limited condition. Therefore, adaptive head lamps (AHL) have been developed to solve the problem^17–20^. In an AHL, the illumination area can be optimized for the driver and people ahead of the driven vehicle as well by switching the light sources including laser diodes or LEDs^21–26^. In this paper, we will present a study of a head lamp with a reflector and a mini-LED matrix to perform low beam and high beam in various illumination ranges. Besides, we first propose to emit high beam with infrared (IR) light by use of IR emitter based on GaN die on the same LED platform. The IR high beam will not induce glare to people ahead of the driven vehicle and has no light pollution to the driving circumstance. The most important point is that the IR high beam can be detected by a photo imager, and so it will be helpful for an autonomous vehicle without use of an AHL.

## Optical design

Reflectors are usually used to project low beam or high beam rather than to project an adaptive head light because the reflector is used for nonimaging projection. A design with a way of light field technology has used a single reflector to perform low beam and high beam with two LED modules where three LED dies are composed in each module. Owing to the constraint of the used LED module, the illumination area/range is not adjustable. This property cannot provide feasible lighting according to different roadway conditions. Therefore, the design in this paper must be divided into two parts, i.e., the light source and the reflector.

**Figure 1:**
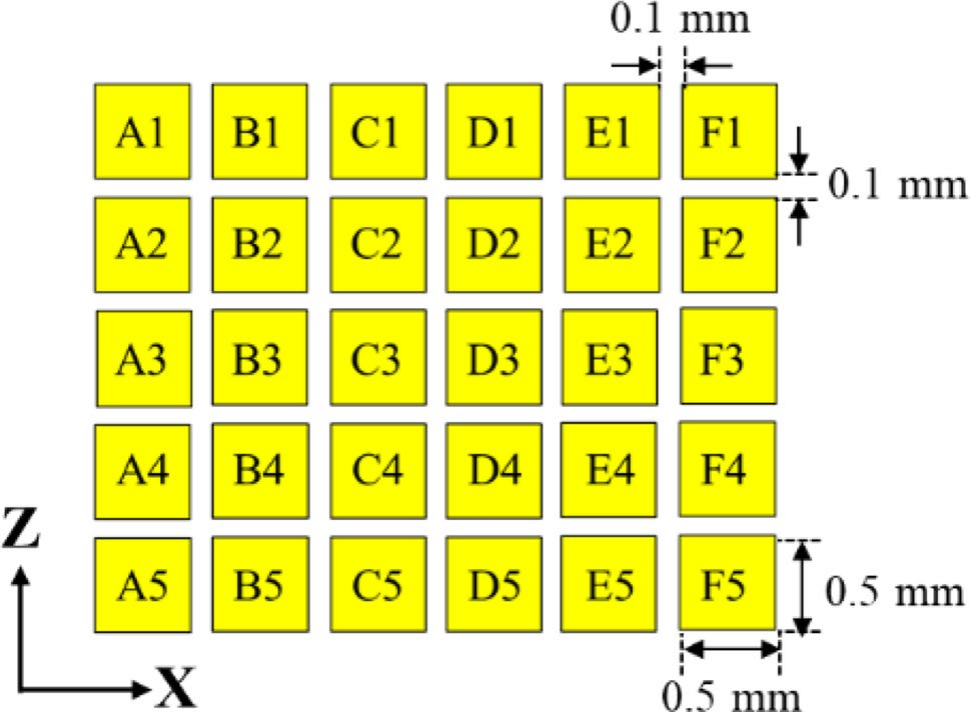
The detailed arrangement of the mini-LED matrix.

**Figure 2:**
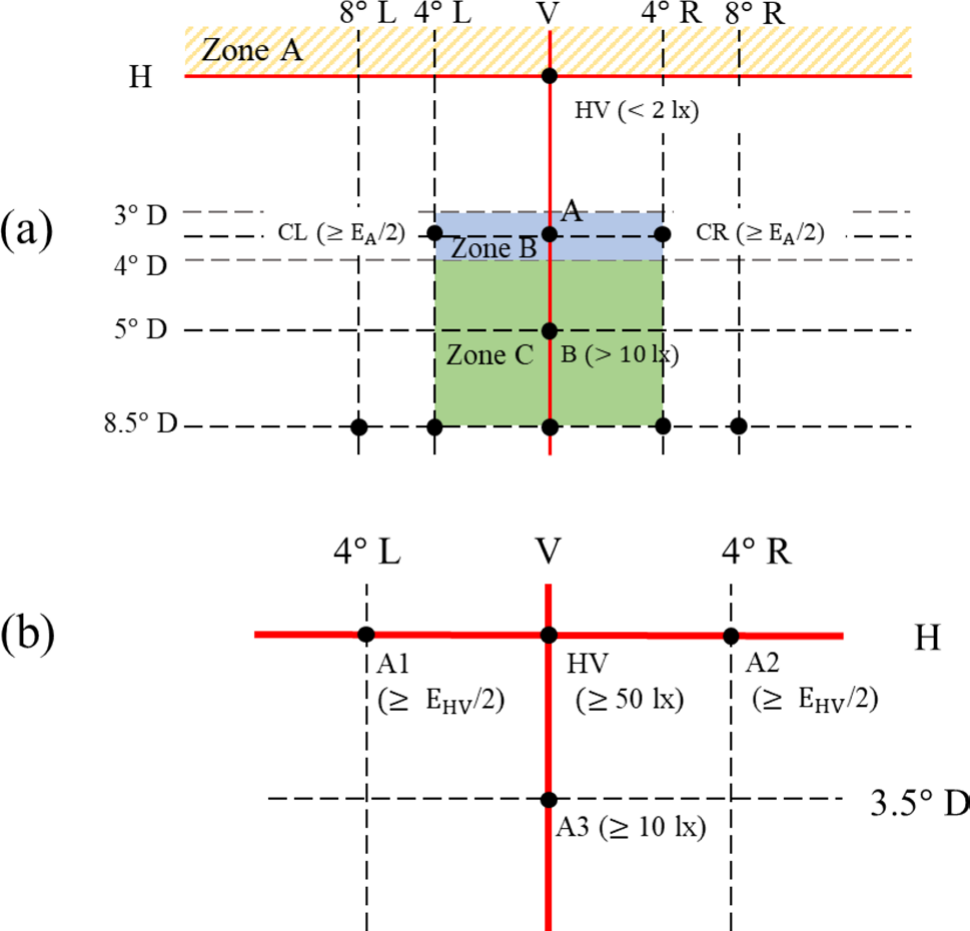
The K-mark regulation for (**a**) the low beam and (**b**) the high beam.

In regarding the light source, to ensure the maximum feasibility of illumination range and reduce the cost, we select the LED die with dimensions of 500 μm × 500 μm, which are the maximum size ranked as mini-LEDs^27^. To reduce the cost, all the emitters are GaN blue dies so that the die bonding, phosphor coating and electrical driving will be consistent. To make the light source as tight as possible and the adjustable range as wide as possible, we design a light source with a 5 × 6 matrix as shown in Fig. 1. To simplify the technical instruction, we choose the K-mark regulation as the reference for the head lamp^28^. In the regulation, there are several check points for low beam and high beam, as shown in Fig. 2, where A point is the main bright point for the low beam, and the ratio between the illuminance at A point (E_A_) and at HV point in the dark zone (must be less than 2 lx) is defined as the contrast, which must be larger than 20. In the high beam, HV is the main bright point, and the illuminance at this point must exceed 50 lx.

In the previous study, the LED die with the area of 1 mm^2^ is powerful enough to support the low beam or the high beam^29^. Thus, at least four LED dies in the mini-LED matrix should be used for supporting the low beam or the high beam. In the design, the LED dies in the bottom two rows are designed for the low beam and the upper three rows are for the high beam.

**Figure 3:**
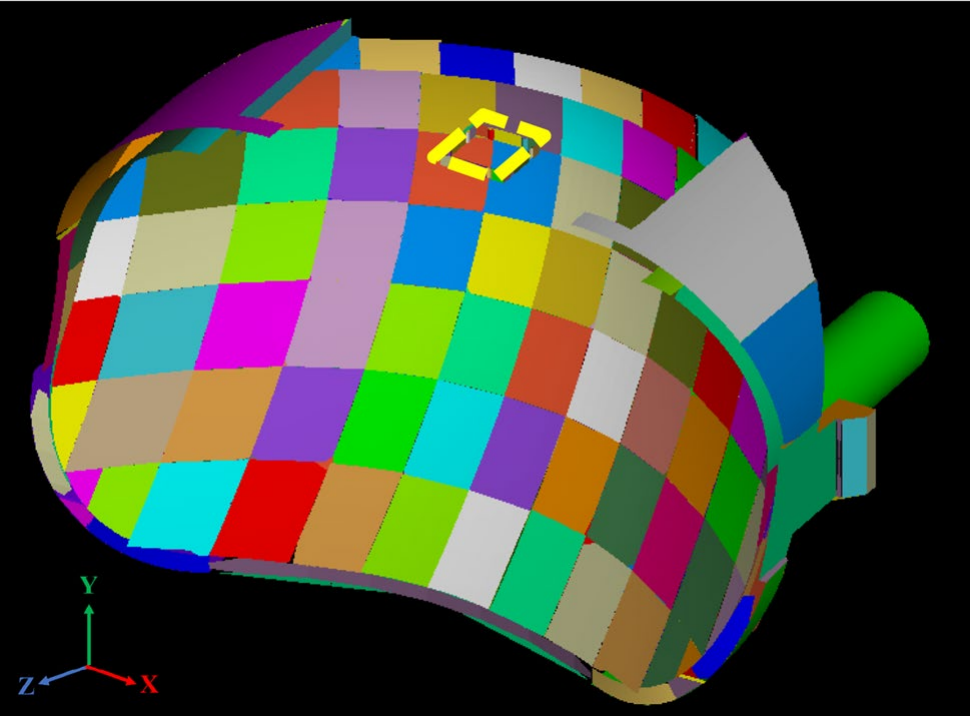
The geometry of the reflector with 68 segments.

The optical design of the reflector should take care of the low beam and the high beam with the designed mini-LED matrix. We set the dies numbered C4/D4/C5/D5 as the main light source for the low beam (so-called L-group), and B2/C2/D2/E2 for the high beam (so-called H-group). The reflector was divided into 68 segments shown in Fig. 3. The optical design follows the rule of light field technology^30^. Since the etendue of the light source received by each segment is different, the specific segments (called HL segments) with smaller etendue are used to project low beam with a high-contrast cutoff line, and the other segments are projected downward for ground illumination. The LED dies responsible for the high beam are far from the reflector, so the reflected rays from the HL segments will propagate upward to form the high beam. Thus the HL segments are for both the low beam and the high beam, and the difference is made by the location of the LED dies. Besides, the mini-LED matrix contains more LED dies along lateral extension other than the L-group and H-group. The result is that the light pattern will be wider when these LED dies are turned on, and this property could be used to extend the illumination area and provide wider vision.

**Figure 4:**
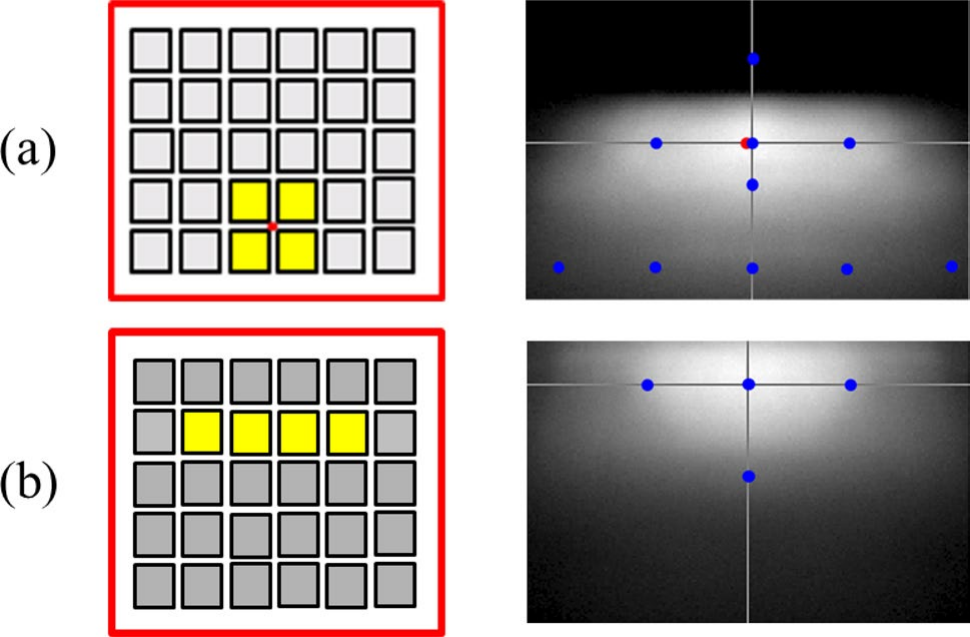
The turned-one LED dies (yellow) at different locations and the corresponding light patterns, where (**a**) is for low beam, and (**b**) is for high beam.

In the design, four die of the mini-LED matrix as a unit light source was used to provide sufficient flux for the light pattern of K-mark low beam. The dimensions of each LED die was 500 μm × 500 μm. The out flux of each miniLED was set 50 lm, which was the average flux of each die obtained by measurement. The simulation was made by using ASAP for Monte Carlo ray tracing^31^. The top surface of each LED die was set Lambertian surface owing to heavy scattering of the phosphor layer^32,33^. The reflector is shown in Fig. 3, where the dimensions of the height, width and depth were 20 mm, 44 mm, and 19.76 mm, respectively, and the reflectivity was set 80%. Figure 4 shows the simulation of the illuminated patterns with different LED dies at 10 m away from the reflector. The observation plane covers an area of 4 m × 2.5 m. The simulation results are shown in Fig. 4. Figure 4a shows the low beam by 2 × 2 array. The illuminance at A point in the regulation was 84.7 lx, and the illuminance at HV point is lower than 2 lx. Figure 4b shows the high beam by 1 × 4 array, which was used to extend light pattern laterally. The illuminance at HV point in the regulation was 90.4 lx, which was larger than 50 lx as requested in the regulation. All the light patterns were examined and met the requirement of the K-mark regulation.

## Experimental measurement

**Figure 5:**
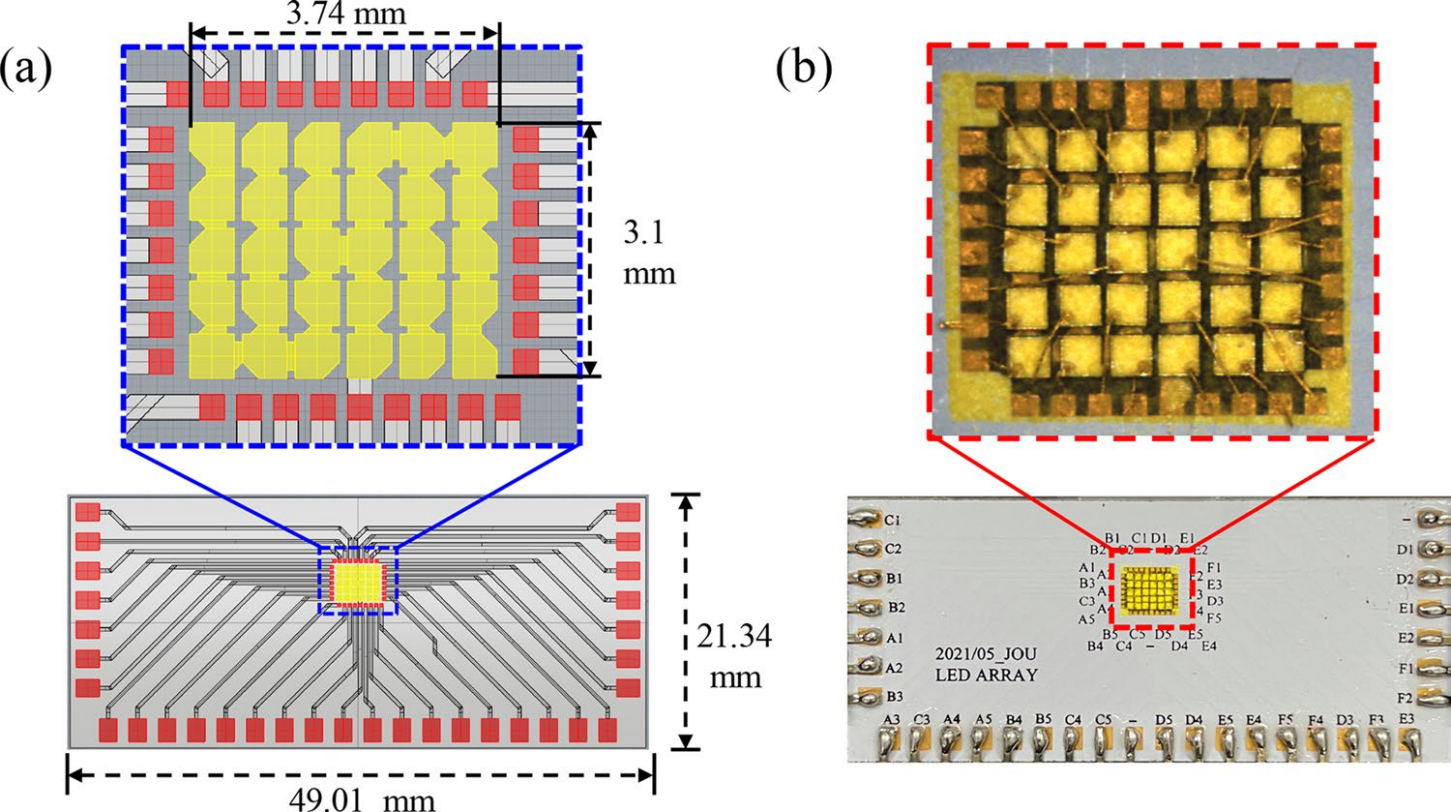
(**a**) The designed arrangement of the LED dies and the MCPCB layout. (**b**) The corresponding sample.

**Figure 6:**
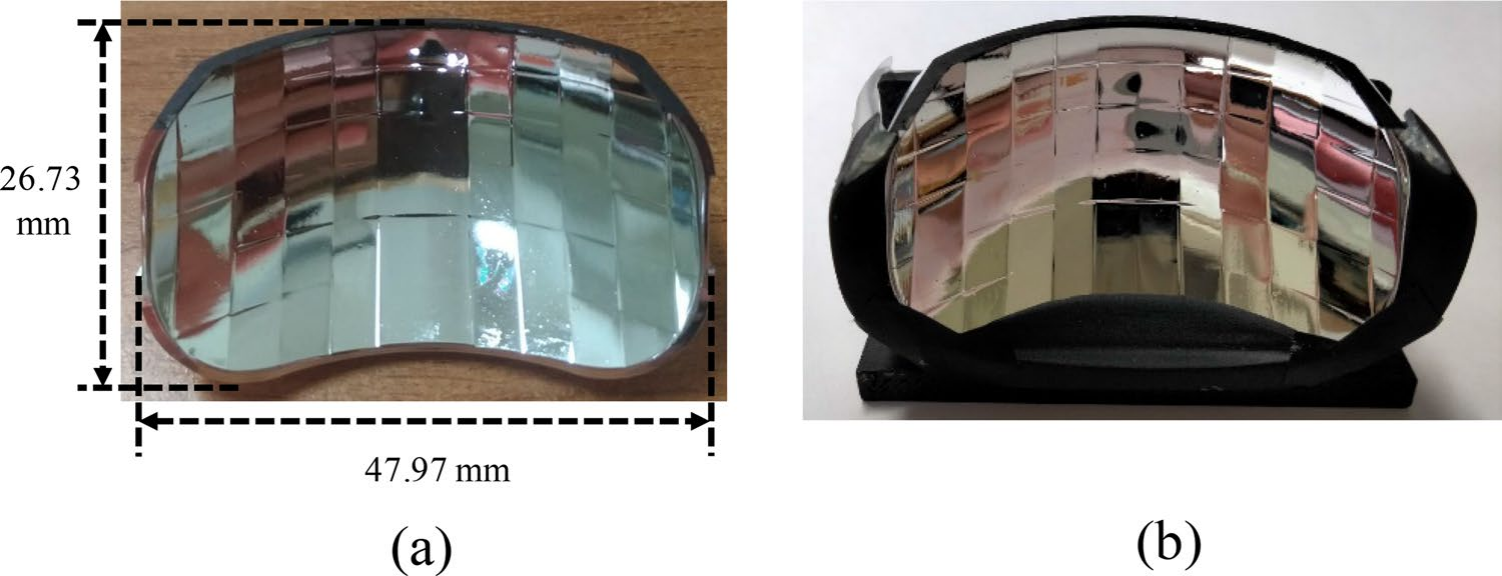
(**a**) The sample of the designed reflector, and (**b**) with a holder in the experiment.

The experiments were conducted in various ways. First, we designed the LED board shown in Fig. 5, where the 30 LED dies by Osram were all in vertical structure and were covered with YAG phosphor after die bonding. The electrical injection was with common cathode, and so any LED could be turned on independently. The reflector was made through CNC machining on a plastic bulk medium and the surface was polished and coated with aluminum film, as shown in Fig. 6.

**Figure 7:**
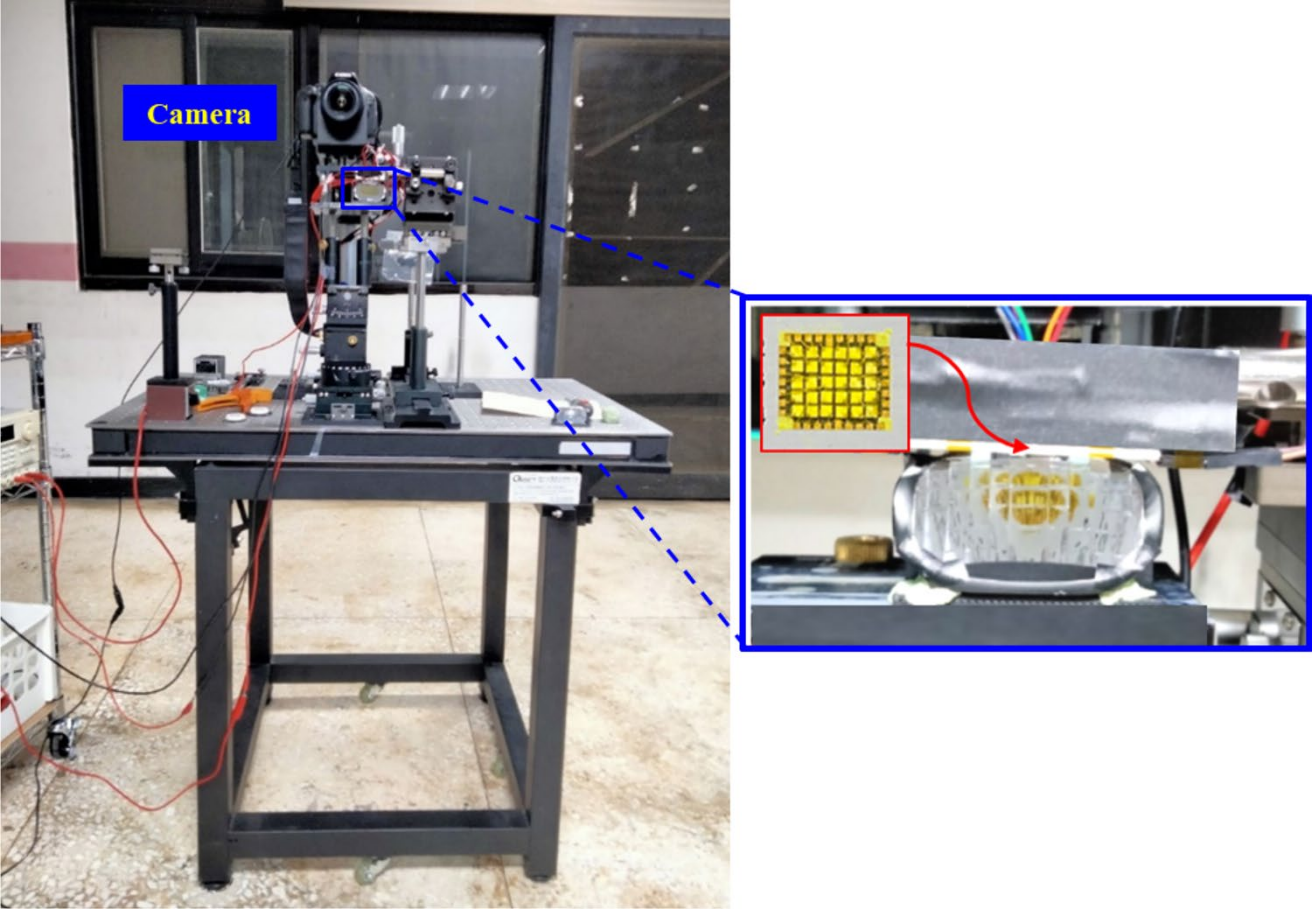
The head lamp module on the test platform in the experiment.

**Figure 8:**
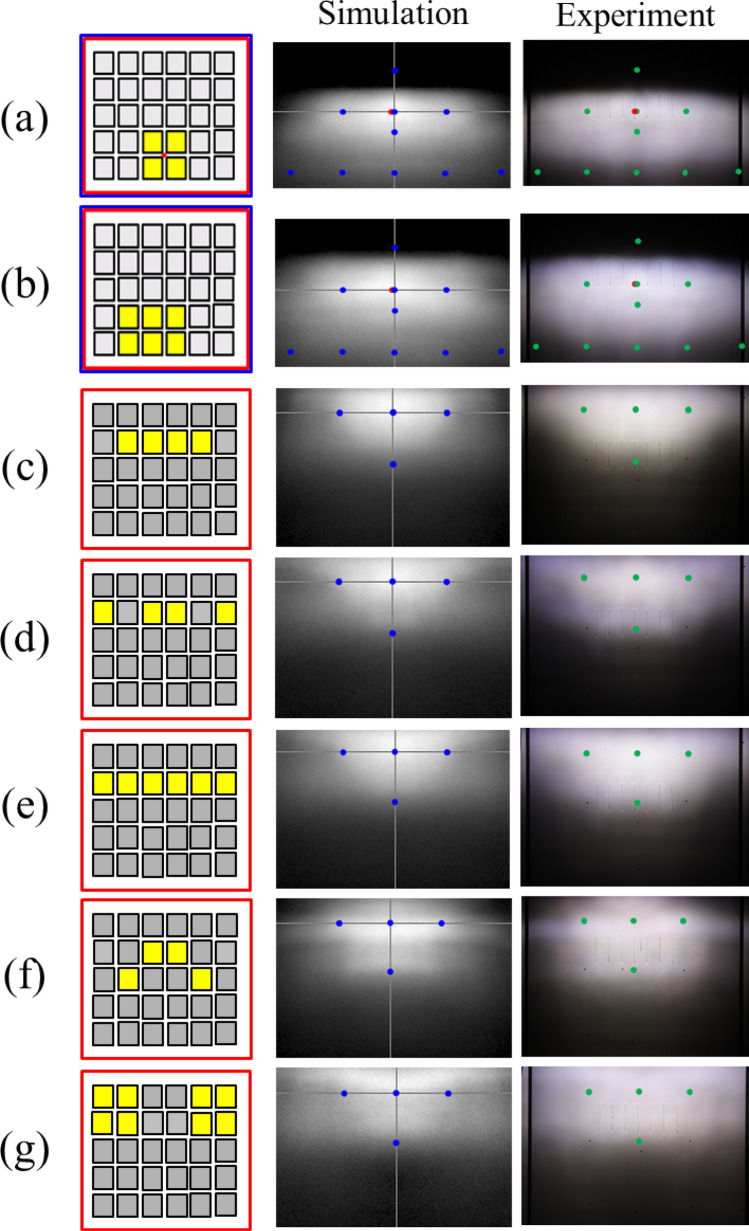
The simulated and observed light patterns and the corresponding turn-on LED dies for (**a**,**b**) low beam, and (**c**–**g**) high beam.

**Figure 9:**
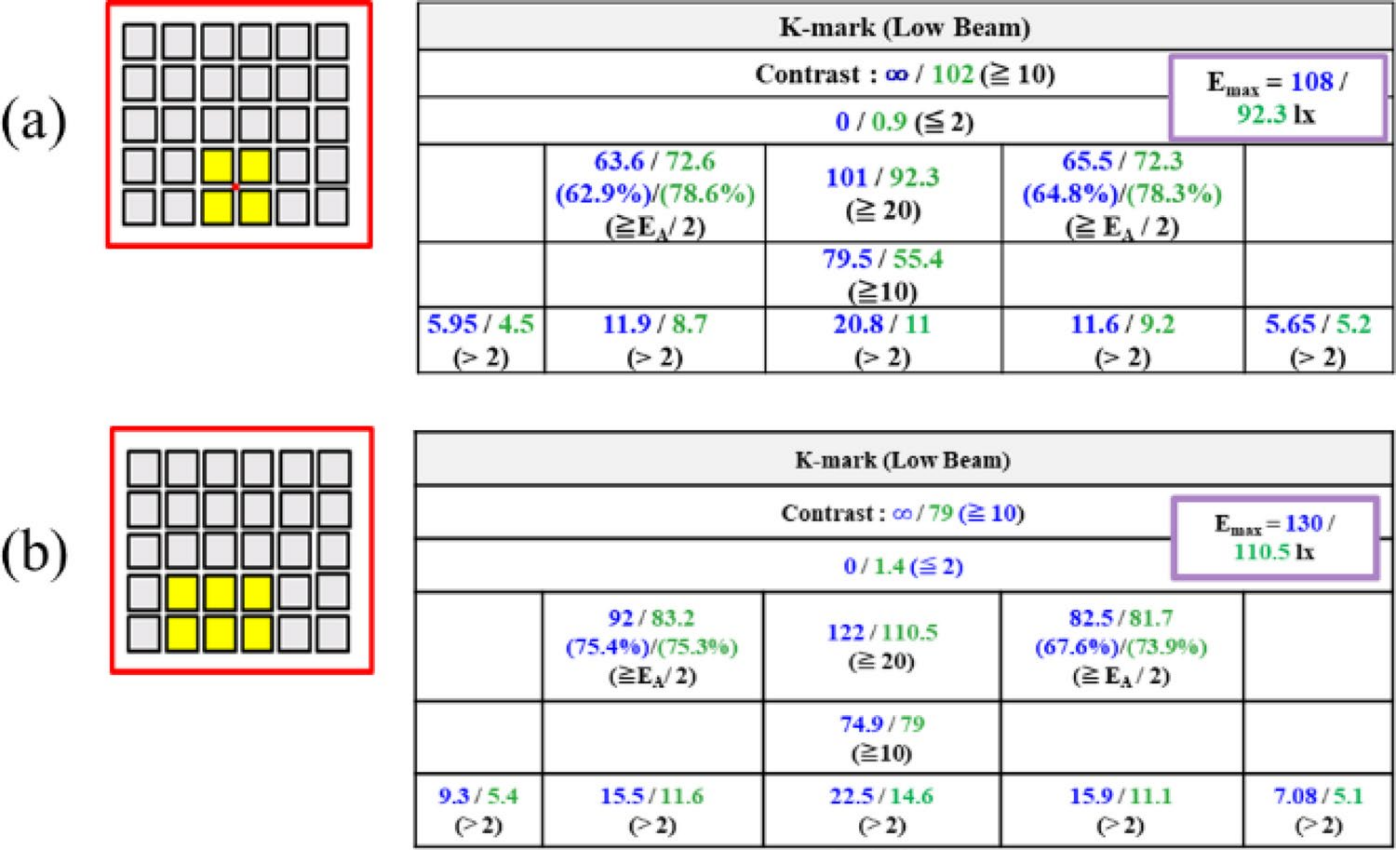
The check points for the low beam of the K mark regulation when (**a**) C4/D4/C5/D5 and (**b**) B4/B5 and C4/D4/C5/D5 were turned on.

**Figure 10:**
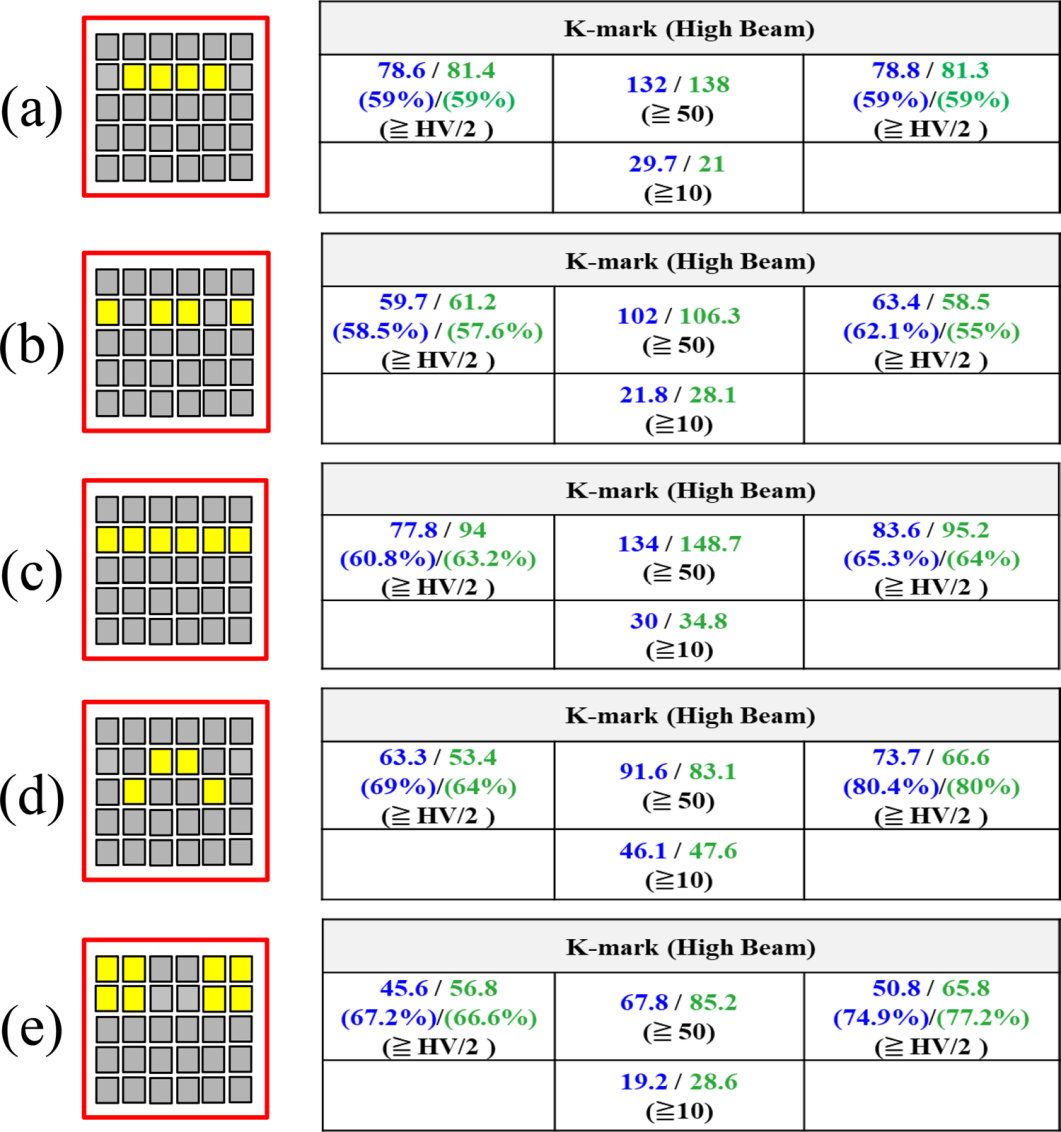
The check points for the high beam of the K mark regulation when (**a**) B2/C2/D2/E2 (**b**) A2/C2/D2/F2 (**c**) A2/B2/C2/D2/E2/F2 (**d**) B3/C2/D2/E3 (**e**) A1/B1/E1/F1 and A2/B2/E2/F2 were turned on. The first value in blue is from the simulation, and second value in green is by measurement.

In the experiment for testing the illumination pattern, the LED board was attached on the top face of the reflector, as shown in Fig. 7. The headlamp module was mounted on a test platform, and was 1.2 m above the ground. A digital imaging system was used to detect the illuminance on the wall at a distance of 10 m away from the headlamp module. The observed illumination patterns on the wall are shown in Fig. 8, where all the light patterns passed K-mark regulation when the driving current was 350 mA for each LED die in the turn-on state, and the average luminous efficacy was around 85 lm/W. The detailed measurement data of the illuminance for the K mark regulation is shown in Figs. 9 and 10, where the measurement showed that the K-mark regulation can be met in all cases. The error of the exact values in Fig. 10 could be caused by the setting flux of the LED mini-LED dies. The smaller error of the ratios are a more important factor to show the design validity. The results also indicated that the optical design rule was applicable to using a mini-LED matrix as the light source. Besides, there were various selections of the turn-on mode for both the low beam and high beam. More turn-on LED dies will laterally extend the illumination pattern and provide wider vision to the user.

## Infrared HIGH beam

The mini-LED has been shown the feasibility in performing various illumination patterns through a single reflector. Here we try to extend the application to forward lighting through a simple change on the mini-LED matrix. The AHL is the new trend in vehicle forward lighting. The main advantage of an AHL is adjustable in the illumination pattern including dimmable spots^34,35^. Such a property allows free of glare when using the high beam on the roadway, and the driver can have a wider and clearer vision. However, an AHL system needs complicated image sensing and image processing. It makes the head lamp more expensive. If a head lamp can project high beam with infrared light for machine vision, it will not induce glare to people ahead of the driven vehicle. This condition is applicable to an autonomous vehicle because machine vision is responsible for driving a vehicle.

**Figure 11:**
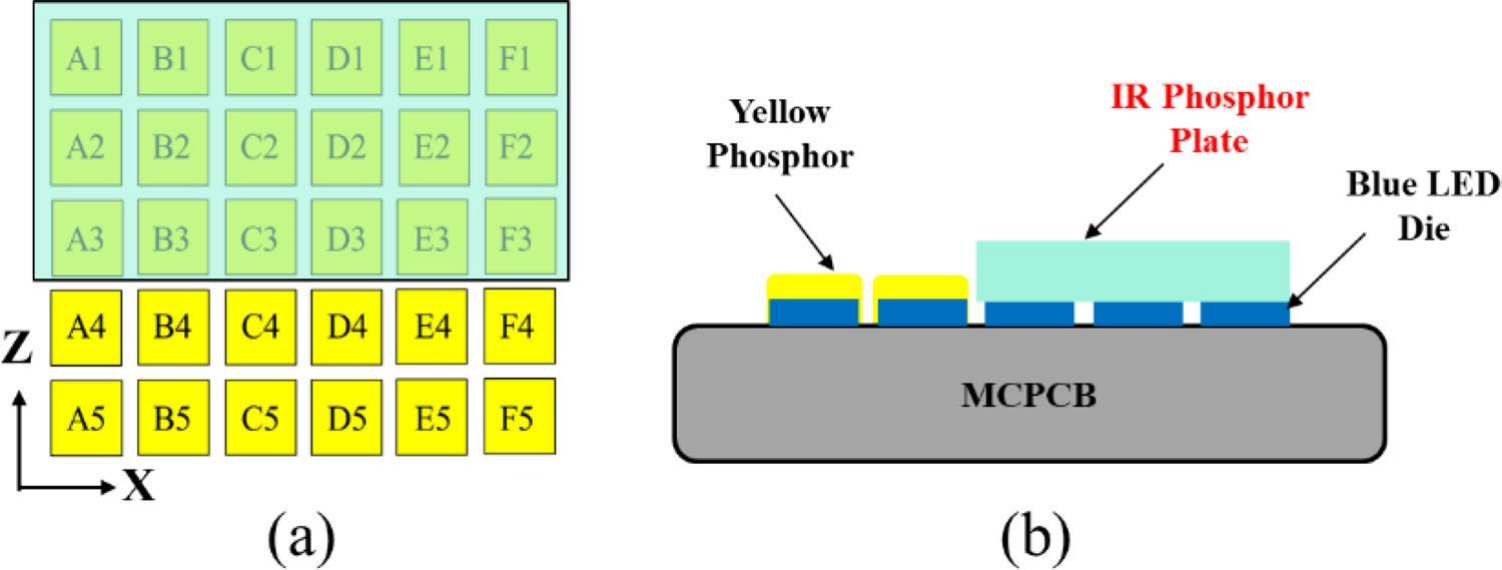
The schematic diagram of the LED matrix with yellow and IR phosphors. (**a**) The top view, and (**b**) the side view.

Therefore, in this section we will present the study of the mini-LED matrix with emission of IR light. Owing to the problem of die bonding and electrical driving, incorporating IR dies on the MCPCB with GaN die could not be a low-cost option. However, here the only change to the mini-LED matrix is to dispense IR phosphor on some GaN dies corresponding to the high-beam projection. Figure 11 shows the concept, where the LED dies for the low beam are covered with yellow phosphor to emit white light, and the others are covered with IR phosphor.

**Figure 12:**
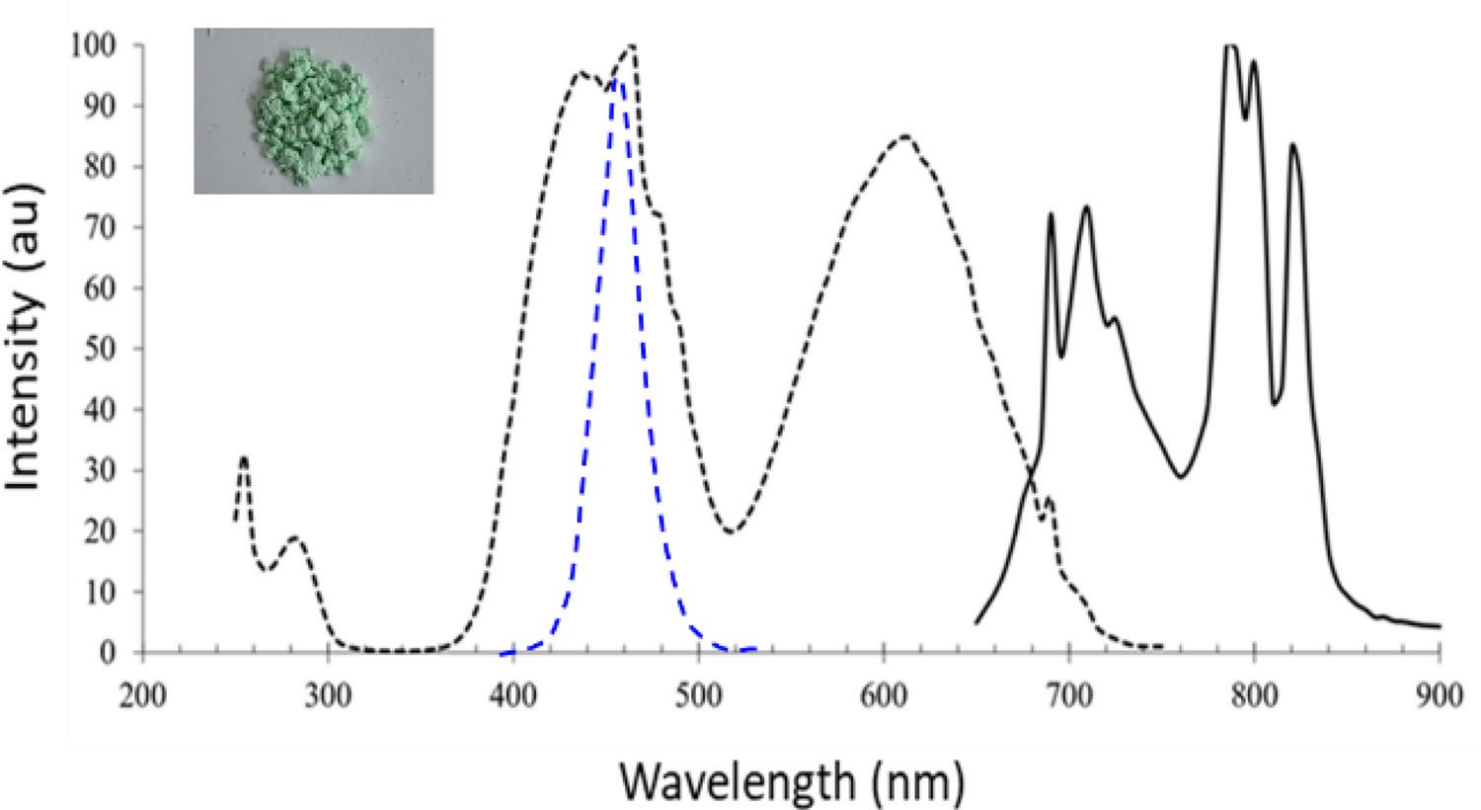
The excitation and emission spectra of the IR phosphor. The blue dash line is the measured spectrum of a blue die.

The use of IR phosphor with blue die could bear large Stokes loss owing to large difference between the pumping and emission wavelength^36,37^. However, GaN blue dies have been well developed, and the external quantum efficiency could reach 80%^37^. Thus the larger Stokes loss could be compensated through high performance of blue dies. In the study, YAGG: Cr, Nd, Eu-based IR phosphor was adapted, where the excitation and emission spectra are shown in Fig. 12. The excitation spectrum covers two peaks in blue light and red light. The blue band coincides with the spectrum of the blue die so that the excitation rate could be high. However, red LED dies are also with higher excitation rate with an additional benefit of lower Stokes loss, and so we could examine the radiant efficiency with the red LED dies in comparison with the blue LED dies.

**Figure 13:**
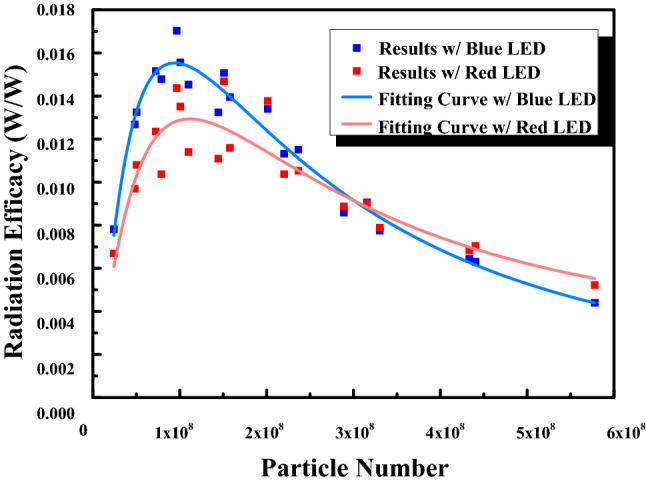
The radiant efficiency with respect to particle for blue and red pumping.

**Figure 14:**
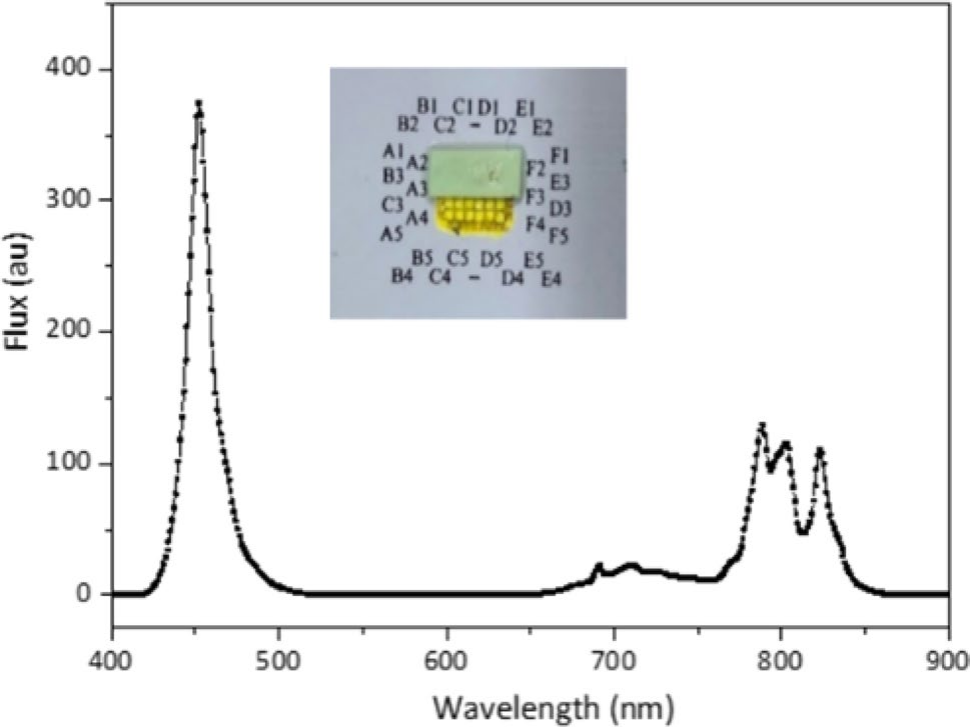
The measured spectrum by turning on the LED dies in the second row, which was covered with the IR phosphor.

To optimize the emission efficiency of the IR phosphor, various phosphor plates were prepared. Those phosphor plates were made with silicone mixed with IR phosphor. We adjusted the thickness and the phosphor concentration of the phosphor plate and measured the IR light flux pumped by a GaN LED die and a red LED die at the peak wavelength around 450 nm and 632 nm, respectively. To reduce the factor of the recycled photons from the backward IR emission, there was a long black tube between the pumping LED and the phosphor plate. The flux by the LEDs were measured by an integrating sphere. The flux of the emission light passing the IR plate was measured by the same integrating. From the measured spectrum, we could obtain the IR light flux. The optimized recipe can be interpreted with the particle number of the phosphor shown in Fig. 13^38^, where we found that the blue pumping light was better than red pumping light. There was not only one reason to cause this result, and both the radiant efficiency were low. We left these issues for the future study. After optimizing the phosphor plate recipe, the particle number for the best recipe is too large to be a film in our process. Thus we used a phosphor plate instead of a heavy film to cover the blue dies located on the upper three rows, which were responsible for emitting the high beam. The measured spectrum is shown in Fig. 14.

**Figure 15:**
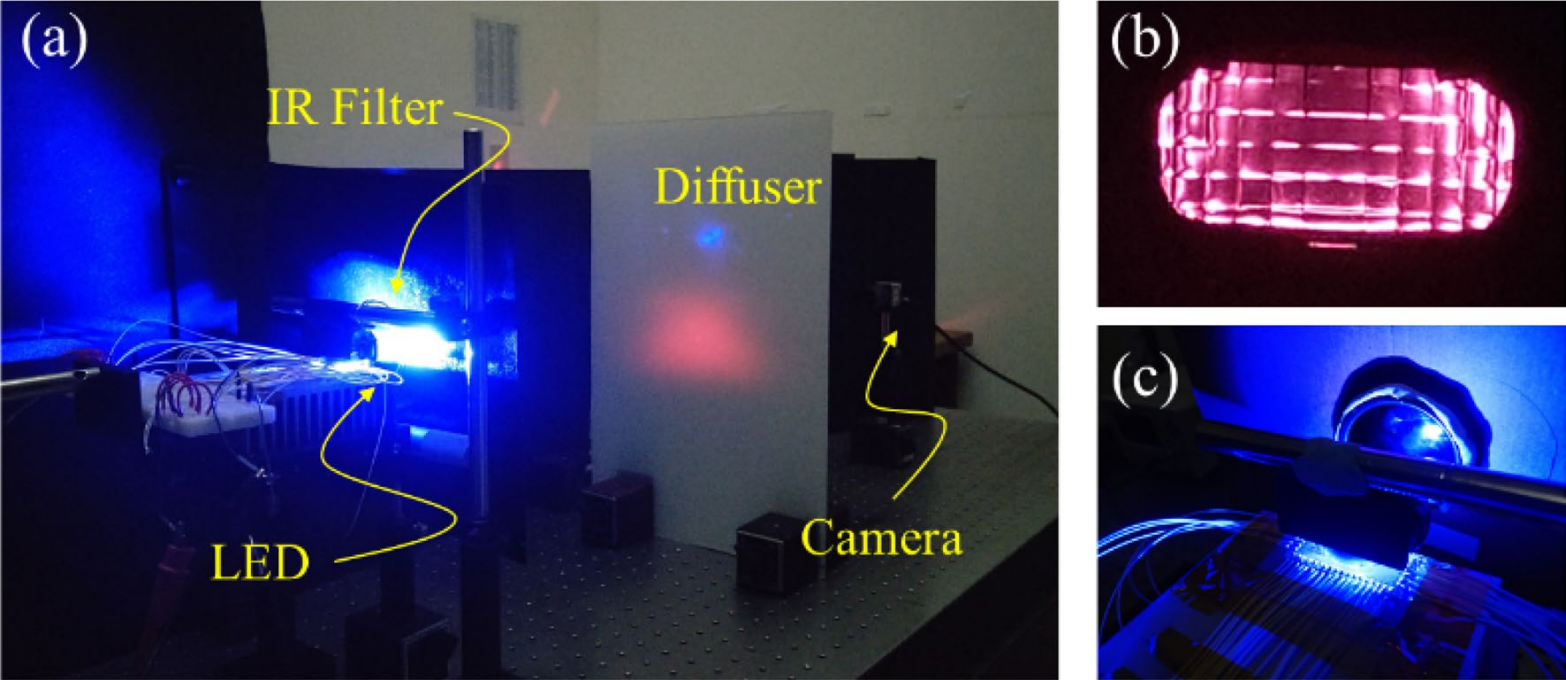
(**a**) The experiment setup, (**b**) viewing the reflector through the IR filter, and (**c**) viewing the IR filter from the LED side. The light pattern is up side down in the experiment owing to the setup geometry.

**Figure 16:**
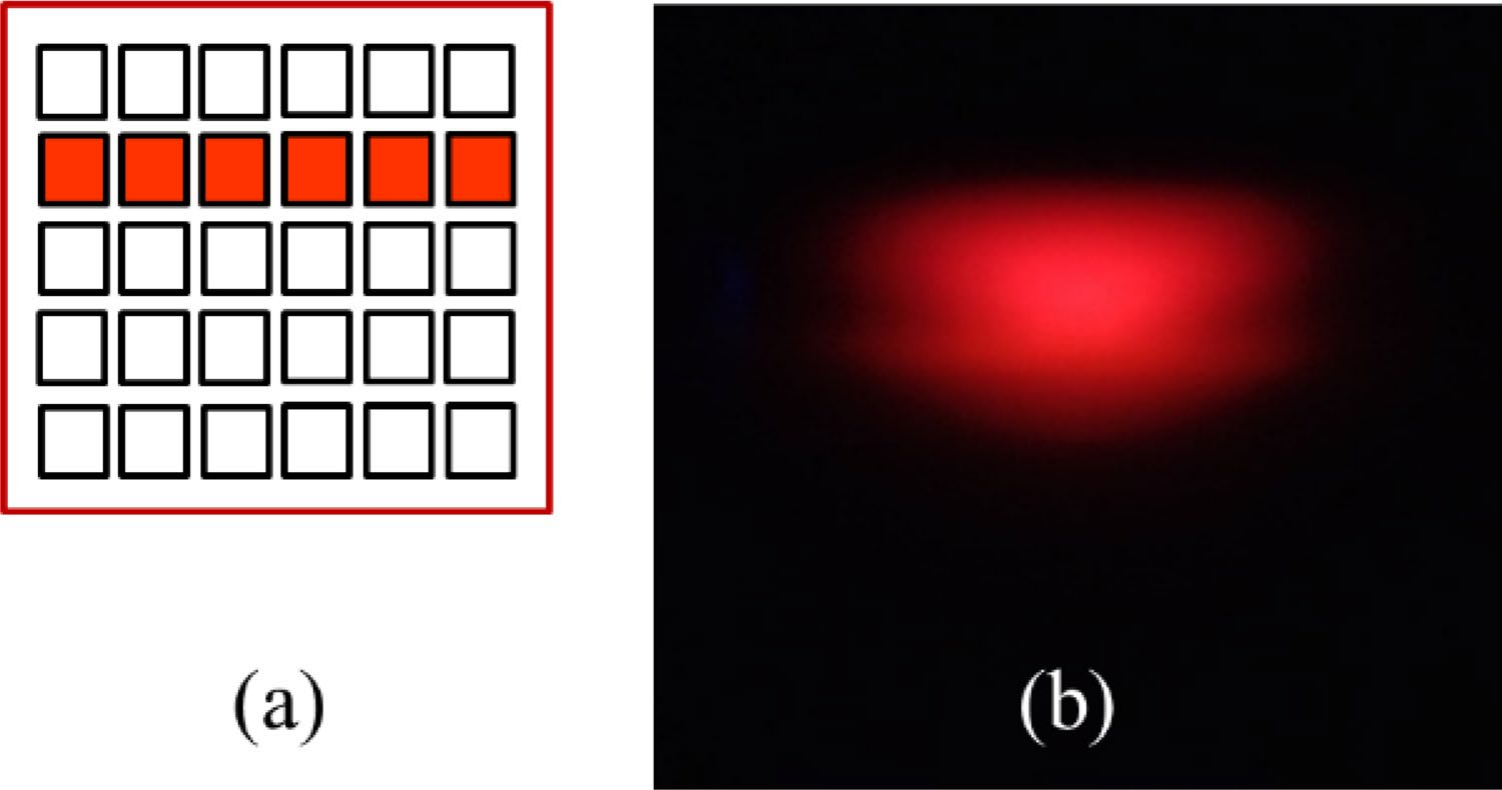
(**a**) The locations of the turn-on LED dies, and (**b**) the observed light pattern by the CCD camera with an IRLP filter at 720 nm.

To visualize the IR illumination pattern, an IR low-pass (IRLP) filter to filter out all the visible light could be used. However, to enhance the visualization, we used an IRLP filter with shorter cutoff wavelength at 720 nm in the experiment. Thus the filtered light pattern could be seen in red. The light pattern was incident on a diffuser at a distance of 60 cm from the headlamp module to ensure the observation plane was at the far field region of the reflector. Behind the diffuser, there was a CCD camera used to catch the illumination pattern, as shown in Fig. 15. We observed the light pattern in red with the IRLP filter at 720 nm and in IR with the IRLP filter at 850 nm, and found no difference in light pattern. Therefore, we show the light pattern in red for convenience. The LED dies in the second row were turned on and the light pattern shown in Fig. 16 was observed, which was similar to that in Fig. 8e, and it meant that the IR light was successfully projected to form the high beam through this way.

## Conclusion

In this paper, we first designed and demonstrated a single reflector for projection of the low beam and high beam with a mini-LED matrix as the light source. Based on the previous reports, the configuration of the mini-LED matrix was designed as 5 × 6, where the bottom 2 rows were for the low beam and the others were for the high beam. Through the light field management, the segments at the reflector with lower etendue were designed to project the low beam with high-contrast cutoff line for the LED dies in the bottom 2 rows, and to project the high beam for the LED dies in the other area. The simulation and experimental measurement shows that the design was successful and applicable. The mini-LED matrix provided a flexible light source to project the low beam and the high beam. Moreover, the illumination pattern could be laterally extended when more LED dies were turned on, and this made the illumination pattern adjustable.

To extend the usage of the mini-LED matrix, we proposed to coat IR phosphor on the blue LED dies in the high-beam zone. What we chose for IR light emission was the phosphor based on YAGG: Cr, Nd, Eu, with two main excitation bands, i.e., a blue band and a red band. Through precise experiment for recipe optimization, higher radiant efficiency could be obtained with the blue dies, and this characteristic fit well with the mini-LED matrix with blue dies. The high beam pattern by IR light was observed similar to that by white light through the same reflector. The IR high beam projected through the proposed mini-LED matrix platform will be useful for machine vision in an autonomous vehicle.
